# Epigenetic Dysregulation in Laryngeal Squamous Cell Carcinoma

**DOI:** 10.1155/2012/739461

**Published:** 2012-05-07

**Authors:** Thian-Sze Wong, Wei Gao, Zeng-Hong Li, Jimmy Yu-Wai Chan, Wai-Kuen Ho

**Affiliations:** Department of Surgery, Queen Mary Hospital, The University of Hong Kong, 102 Pokfulam Road, Hong Kong

## Abstract

Laryngeal carcinoma is a common head and neck cancer with poor prognosis. Patients with laryngeal carcinoma usually present late leading to the reduced treatment efficacy and high rate of recurrence. Despite the advance in the use of molecular markers for monitoring human cancers in the past decades, there are still no reliable markers for use to screen laryngeal carcinoma and follow the patients after treatment. Epigenetics emerged as an important field in understanding the biology of the human malignancies. Epigenetic alterations refer to the dysregulation of gene, which do not involve the alterations of the DNA sequence. Major epigenetic changes including methylation imbalance, histone modification, and small RNA dysregulation could play a role in the development of human malignancies. Global epigenetic change is now regarded as a molecular signature of cancer. The characteristics and behavior of a cancer could be predicted based on the specific epigenetic pattern. We here provide a review on the understanding of epigenetic dysregulation in laryngeal carcinoma. Further knowledge on the initiation and progression of laryngeal carcinoma at epigenetic level could promote the translation of the knowledge to clinical use.

## 1. Introduction

Head and neck squamous carcinoma is estimated to be the sixth most common malignant tumor worldwide. Of which, laryngeal carcinoma is the second most common head and neck squamous carcinoma [1]. According to the global cancer statistics in 2008, the age-standardized incidence rate (ranged from age 0–74) is 5.5 per 100,000 in men and 0.6 per 100,000 in women in developed areas; in less developed area, the incidence rate is 3.5 per 100,000 in men and 0.6 per 100,000 in women [2]. It is estimated that there are 12,740 new patients (male 10,160 and female 2,580, resp.) suffering from laryngeal carcinoma in the United States in 2011 (accounting for 0.7% of the total new cancer cases). It also accounts for about 0.6% estimated cancer-related deaths in the United States [3]. In Hong Kong, laryngeal carcinoma took the third place in the incidence of the head and neck cancer in 2009 [4].

Most of the laryngeal carcinoma patients are male [5]. Moreover, the majority of the patients lie in the middle-aged group [6]. The definite cause of laryngeal carcinoma is not yet determined, while some risk factors are believed to be linked with the development of the disease. Tobacco and alcohol consumption are the primary aetiologic factors [6–8]. Chronic laryngeal inflammation induced by irritants and prolonged voice abuse could also contribute to the development of laryngeal carcinoma [9]. Recently, it was demonstrated that viral infection is a plausible cause for laryngeal carcinoma. However, the causal link between HPV and laryngeal carcinoma remains controversial. Human papilloma virus (HPV) types 6, 11, 16, 18, and 33 are detected in patients with laryngeal carcinoma [10]. Among all the detected HPV subtypes, the infection prevalence of HPV type 16 is much higher than the infection of the other types [11]. The link between HPV infection and laryngeal carcinoma development is affected by geographical factor and was confirmed in two large case-control studies [12].

Diagnosis of laryngeal carcinoma depends on the following: symptoms (e.g., hoarseness, voice changes, and feeling of a lump in the throat); signs (e.g., neck mass and laryngeal tenderness); assistant examination (including CT scan, electronic laryngoscopic detection, and pathological biopsy) [13]. Among these, the result of pathological biopsy, which can supply a histological evidence, is the golden standard for the diagnosis of laryngeal carcinoma. Histologically, most of the laryngeal carcinomas are squamous cell carcinoma. Morphological changes including dyskeratosis, laryngeal intraepithelial neoplasia, atypia, and dysplasia are lesions with high chance to transform from mild dysplasia to carcinoma in situ [14]. In comparison, minor squamous epithelial changes such as squamous metaplasia, squamous hyperplasia, pseudoepitheliomatous hyperplasia, keratosis, and parakeratosis may be found in the laryngeal region without subsequent malignant transformation [14].

In order to find out the definite cause of laryngeal carcinoma and the available index for the diagnosis of this disease, researchers put their attention on the research of molecular markers. Since 1983, the epigenetics of human cancer draw the researchers' attention [15]. It is now recognized that epigenetic markers including hypermethylated DNA and oncogenic microRNA could be used as screening markers for human cancers. Further, epigenetic drugs including decitabine, zebularine, and TSA are under examination for use as anticancer drug by reversing the altered epigenetic traits of cancer cells. In comparison with the epigenetic studies in other solid cancers, studies on laryngeal carcinoma are comparatively fewer. In this paper, we summarized the current knowledge on epigenetic alterations in laryngeal carcinoma from the published reports.

## 2. Epigenetic Alterations in Human Malignancies

Wu and Morris defined epigenetics changes as “changes in gene function that are mitotically and/or meiotically heritable and that do not entail a change in DNA sequence” [16]. Thus, an epigenetic trait is developed from the epigenetic changes in gene expression without the alteration of DNA sequence [17]. In general, epigenetic alterations broadly cover all the changes resulting in gene expression regulation without interfering with the DNA sequence at genetic levels [18]. In human cancers, common epigenetic alterations include DNA methylation, histone modification, and noncoding RNA dysregulation [14, 15]. In the past decade, studies on the abnormal epigenetic changes involved in human malignancies are numerous leading to the development of epigenetic markers and therapeutic targets at epigenetic level.

### 2.1. Aberrant DNA Methylation Changes in Human Cancers

In 1969, Griffith and Mahler proposed that modification of DNA base is a possible way to modulate gene expression [19]. In mammalian cells, modification of the cytosine residue is the predominant DNA modification, especially in the cytosine of CpG dinucleotide [20]. In human genome, the CpG dinucleotides are particularly rich in large repetitive sequence (e.g., centromeric repeats) and gene regulatory regions (e.g., CpG islands). Aberrant CpG island methylation is catalyzed by mammalian DNA methyltransferase and is usually found in the upstream region of the transcription start sites. CpG island methylation could block the accessibility of transcription factor to the promoter region of the tumor suppressor genes [21]. Additionally, the methylated sequence itself could recruit histone-modifying protein which induces gene silencing by changing the chromatin structure from active open chromatin structure to condensed closed chromatin structure [22, 23]. Approximately, 60% of the protein-coding genes in the human genome contain the CpG islands at their regulatory regions [24]. In cancer cells, CpG islands become methylated and there is de novo addition of methyl group to the C5 position of the cytosine ring [25]. DNA methylation is the most studied epigenetic change in head and neck cancers. In head and neck cancer, aberrant promoter methylation has been found in a wide variety of genes including p14ARF, p15, p15INK4B, p16, p16INK4A, ATM, DCC, DAPK, MINT1, MINT2, MINT27, MINT31, RARbeta, CDH1, cyclin A1, cytoglobin, RASSF1A, LHX6, MLH1, MGMT, and CDKN2A [26–35]. These genes are involved in cell cycle, apoptosis, angiogenesis, cell-cell adhesion, migration, invasion, and metastasis [26–35]. Most of the studies focusing on the head and neck cancers employed a collection of tissues collected from different head and neck regions including nasopharynx, oral cavity, larynx, and hypopharynx. It is difficult to specify a methylation pattern which belongs to the laryngeal carcinoma alone. Here, we performed a review of the methylated genes with clearer association with laryngeal carcinomas.

#### 2.1.1. Chromodomain-Helicase-DNA Binding Protein 5 Methylation

Chromodomain-helicase-DNA-binding protein 5 (CHD5) is involved in modifying chromatin structure. CHD5 protein contains chromatin remodeling, helicase, and DNA-binding motifs. Reduced CHD5 expression has been reported in neuroblastoma [36]. Mutation of CHD5 is a rare event. Loss of CHD5 was linked to the chromosomal deletion on 1p36 and promoter hypermethylation in neuroblastoma [36, 37]. Chromosomal abbreviation at 1p36 has been reported in head and neck carcinoma [38]. Loss of heterozygosity and instability of 1p36 could be detected in primary oral and laryngeal carcinomas [39]. Although loss of CHD5 has been shown in laryngeal carcinoma, the association between 1p36 aberrations and CHD5 loss in laryngeal carcinoma has not yet been confirmed [40]. In laryngeal carcinoma cell lines, CHD5 functions as growth and invasion inhibitor [40]. CHD5 could stabilize the key regulator of head and neck carcinoma, P53, through activating expression of p19arf [41]. Recently, it was shown that promoter region of CHD5 was methylated in laryngeal carcinoma cell lines [40]. The epigenetic changes of CHD5 expression are reversible by demethylating agent. Demethylation treatment of laryngeal carcinoma cell line with 5-Aza-dC could restore CHD5 expression [40].

#### 2.1.2. E-Cadherin Methylation

E-cadherin (cadherin 1, type 1, E-cadherin (epithelial)) is a 97-kDa transmembrane glycoprotein. Functional E-cadherin protein contains five extracellular cadherin repeats, a transmembrane region and a cytoplasmic tail. E-cadherin functions as adhesion protein of epithelial cells and is involved in cell attachment and the cell polarity and tissue architecture [42]. Loss of E-cadherin expression enhances head and neck cancer cell migration and increases the risk of metastasis [43]. Further, loss of E-cadherin could promote proliferation of head and neck cancer cells through activating epidermal growth factor receptor (EGFR) pathways [44]. In laryngeal carcinoma, reduced expression of E-cadherin has also been reported [45, 46]. Loss of E-cadherin is particularly associated with supraglottic carcinoma of the larynx and is associated with the aggressiveness of the cancers [47]. Downregulation of E-cadherin of supraglottic larynx carcinoma is associated with histological differentiation and/or lymph nodes metastases [48]. Loss of E-cadherin is considered as a good indication of regional lymph node metastasis in laryngeal carcinoma patients [49–51]. In addition, the expression level of E-cadherin is a potential indicator to predict the effect of overall treatment time in patients with supraglottic carcinomas [52]. In head and neck cancers, loss of E-cadherin in parallel with promoter hypermethylation is frequently reported and is suggested to be linked with the aggressive behavior of the cancers [47, 53]. In a case control study with 235 patients with laryngeal and hypopharyngeal cancers, E-cadherin was reported as one of the most commonly methylated gene in laryngeal and hypopharyngeal cancers; however, no significant predictive value was found [54]. Recently, Marsit et al. demonstrated that methylated E-cadherin was only associated with head and neck patients who are light smokers [55].

#### 2.1.3. P16 Methylation

P16 or cyclin-dependent kinase inhibitor 2A is a cell cycle regulator controlling G1 to G2 cell cycle arrest. P16 could bind to cyclin-dependent kinase 4 protein (Cdk4) resulting in loss of interaction between Cdk4 and cyclin D1 leading to cell cycle arrest [56]. The expression level of p16 is significantly reduced in laryngeal carcinoma cells [57]. The gene encoding p16 is localized to 9p21, a region where genetic abbreviations including LOH are frequently reported [58]. It has been reported that p16 is subjected to the effects of genetic and epigenetic changes such as allelic loss, point mutations, and hypermethylation leading to the high rate of p16 reduction in laryngeal carcinoma [58, 59]. Concurrent loss of heterozygosity and methylation of P16 has also been reported in laryngeal carcinoma [60]. In comparison with other inactivation mechanisms including homozygous deletion, methylation of the promoter region, and point mutation, p16 methylation tends to be the most frequent inactivation pathways found in head and neck cancer [61]. P16 methylation is a frequent event in laryngeal carcinoma and was detected in more than 80% of the squamous cell cancer of the larynx [62]. Recently, it is demonstrated that methylation of p16 is an early event in squamous cell carcinoma at the laryngopharyngeal region, implying that methylated p16 may have a clinical value in screening patients with early laryngeal carcinoma [63]. Koscielny et al. proposed that methylated p16 had prognostic value in a follow-up period of 3 years [64].

#### 2.1.4. DAPK Methylation

DAPK is one of the most frequently methylated genes in laryngeal carcinoma. Park et al. demonstrated the highest methylation frequencies (87%) in the laryngeal carcinoma tissues [65]. Similar results have been demonstrated by Calmon et al., and they suggested that they observed an additional correlation of methylated DAPK1 with the lymph node metastasis [66]. Death-associated protein kinase 1 or DAPK is a calcium/calmodulin-dependent serine/threonine kinase involved in gamma-interferon-induced programmed cell death. Methylation of DAPK1 is an early event in the carcinogenesis of head and neck cancers [67]. In lung cancers, DAPK methylation was predominant in older patients in comparison with the young [68]. The methylation status of the promoter region of DAPK gene plays a significant part in controlling DAPK transcription in laryngeal carcinoma. In laryngeal squamous cell cancer with DAPK methylation, DAPK mRNA was totally undetectable [69]; methylated DAPK could be employed to be used as a screening marker in head and neck cancers as it could be detected in the saliva of head and neck cancer patients with potential value in predicting cancer recurrence [70]. Later studies confirmed that methylated DAPK is a good minimal invasive biomarker for head and neck cancer screening as it is detectable in the body fluid of head and neck cancer patients [67].

### 2.2. DNA Hypomethylation in Laryngeal Carcinoma Cells

As mentioned above, focal methylation of specific tumor suppressor genes is common in cancer cells. The number of methylated tumor suppressor genes is higher in cancer cells in comparison with the analogous normal counterpart. However, if we look at the density of methylated CpG dinucleotides, there is a substantial reduction of 5-methylcytosine content [71]. The global loss of genomic 5-methylcytosine content is recognized as global hypomethylation and is associated with the dysregulation of DNA methyltransferase DNMT1 [72, 73]. DNMT1 is the most highly expressed methyltransferase in somatic cells [74]. It maintains the 5-methylcytosine patterns on the newly synthesized DNA after cell division.

An example of hypomethylated genes in laryngeal carcinoma cells is S100A4. S100A4 is an acidic calcium-binding protein and functions as oncogene in a variety of human malignancies. In oral squamous cell carcinoma, S100A4 could suppress expression of the epithelial protein E-cadherin [75]. It could also induce angiogenesis through activation of VEGF expression in oral squamous cells [76]. Results from animal models revealed that cancer cells with high S100A4 levels had higher metastatic rate [77]. The aggressive phenotype is partly contributed by the fact that S100A4 could trigger degradation of extracelluar matrix [78]. In laryngeal carcinoma cells, treatment with demethylating agents could induce expression of S100A4 at both RNA and protein levels [79].

The reasons for DNA hypomethylation remain unclear. It is suggested that the global changes in methylation patterns are associated with the dysregulation of the methylation machinery. With a better understanding of the functional role of different DNA methyltransferases, it is now proposed that the loci-specific focal hypermethylation is linked to the dysregulation of de novo methyltransferase 3A and 3B. On the other hand, the global loss in methylcytosine content is resulting from the functional error of DNMT1. Global hypomethylation could lead to activation of oncogenes [80]. It is now recognized that the methylation imbalance resulting from focal methylation and global hypermethylation is the leading cause of inactivation of tumor suppressor genes and activation of oncogenes in cancer cells.

In laryngeal carcinoma, it has been reported that hypomethylation is linked to the polymorphism of the genes regulating DNA methylation including methionine synthase (MTR) and 5, 10-methylenetetrahydrofolate dehydrogenase, 5, 10-methenyltetrahydrofolate cyclohydrolase, 10-formyltetrahydrofolate synthetase (MTHFD1), and methylenetetrahydrofolate reductase (MTHFR). According to Kruszyna et al., patients with 2756AG or GG genotypes on MTR have a higher risk of developing laryngeal carcinoma [81].

### 2.3. The Role of Histone Modification in Laryngeal Carcinoma

Histone is the structural unit of nucleosomes and is important to the packing of DNA. Posttranslational modification of histone (e.g., acetylation, methylation, ubiquitylation, phosphorylation, sumoylation, and ribosylation) could control the activity of the surrounding DNA [82]. Modification on histone protein could affect the protein stability, protein-protein interaction, protein localization, and DNA binding [83]. Laryngeal carcinoma has a higher expression level of H3, however, the modification status of the overexpressed histone is not yet evaluated [84]. Takahashi et al. reported that histone H3; was overexpressed in gastric adenocarcinoma and it was subjected to phosphorylation [85]. Association between histone modification (histone H3 lysine 9 methylation, H3 lysine 4 methylation, H3 lysine 9 acetylation) and the transcriptional regulation of tumor suppressor gene is reported in laryngeal carcinoma [86]. According to the results from Yang et al., histone modification is only a layer of regulation. Histone modification usually works together with other epigenetic mechanisms in controlling gene expression [86].

### 2.4. Interplay between Small Noncoding RNA and Laryngeal Carcinoma

In human genome, the protein-coding portion is only about 2% [87]. The majority of the non-protein-encoding portion is encoding RNA including PIWI-interacting RNAs, small nucleolar RNAs, transcribed ultraconserved regions, and large intergenic noncoding RNAs [88]. According to the size of the noncoding RNA, they could be classified into short ncRNA, midsize ncRNA, and long ncRNA.

### 2.5. Dysregulation of Short Noncoding RNA in Laryngeal Carcinoma

PIWI-interacting RNA, transcription initiation RNA, and microRNA are 3 major small noncoding RNA encoded in the human genome. PIWI-interacting RNA (26–31 bp) was discovered in 2006 and functions through interacting with the Argonaute family protein PIWI [89]. PIWI-interacting RNA could control gene expression by modulating locus-specific methylation [90]. However, its role in human cancers remains to be elucidated. Transcription initiation RNA controls gene expression at postinitiation stage of transcription [91]. It is evolutionarily conserved and is associated with the chromatin state [92].

#### 2.5.1. Candidate Tumor-Suppressing MicroRNA in Laryngeal Carcinoma

In comparison with the other 2 small noncoding RNAs, the pathway of microRNA biogenesis and function in human cancers is much clearer. MicroRNA is small noncoding RNA that regulates gene expression at posttranscriptional level [93]. Mature microRNAs are short single-stranded molecules, which are about 22 nucleotides long. They function by directly interfering with the target mRNA sequence through complementary interaction. The binding of the microRNA to the 3′UTR of the target mRNA will hinder the subsequent translation process or promote cleavage of the target mRNA [94]. It is estimated that microRNA is involved in the control of about 60% protein-coding genes [89].

Head and neck cancer has a distinctive microRNA expression in comparison with their normal counterpart, and the microRNA expression is suggested to play a significant part in the pathogenesis of head and neck cancer [95]. In laryngeal carcinoma, microRNA could be classified into oncogenic microRNA and tumor suppressing microRNA [96]. Tumor-suppressing microRNA should target oncogene under normal circumstance. Lujambio et al. demonstrated that loss/suppression of tumor-suppressing microRNA would promote cancer progression because of the elevation in target oncogene expression [97].

**Let-7**

Let-7 was discovered in 2000 and is one of the well-studied tumor suppressing microRNAs [98]. Let-7 is a family of microRNA including let-7a, let-7b, let-7c, let-7d, let-7e, let-7f, let-7 g, let-7i, let-7b*, let-7e*, and microRNA-98 [99]. Expression changes of let-7 family member have been reported in numerous human cancers suggesting that the let-7 isoform is playing a distinctive function in different cancers. Let-7a could repress RAS and/or c-MYC expression in human cancer cells, and such effects are demonstrated in laryngeal carcinoma recently [100, 101]. Let-7a could sensitize chemoresistant head and neck cancer cells through modulating stemness genes [102]. Let-7d facilitates the development of epithelial-mesenchymal transition (EMT) traits in oral squamous cell carcinoma [103]. In laryngeal carcinoma, let-7 is a growth inhibitor and could induce apoptosis in laryngeal carcinoma cells [100]. Further, let-7 expression is linked to the sensitivity of cancer cells to radio- and/or chemotherapy [104]. It is noted that not all the let-7 family members are downregulated in cancer cells. Let-7i is preferentially upregulated in laryngeal carcinoma, albeit the functional significance remains unclear [105].

**MicroRNA-7**

MicroRNA-7 is a proliferation suppressor [106]. There are reports suggesting that microRNA-7 suppression leads to the upregulation of epidermal growth factor receptor expression [107]. Webster et al. confirm the association, and they observed that microRNA 7 is linked to cell cycle progression and cell viability [108]. In vivo studies demonstrated that microRNA-7 is suppressed by inflammatory response [109]. In addition, increasing microRNA-7 in cancer cell line through liposomal delivery could inhibit cell division and is suggested to be a potential treatment modality [110]. In head and neck cancer, let-7 insulin-like growth factor 1 receptor (IGF1R) pathway is possibly linked with cancer cell migration and invasion [111]. MicroRNA-7 is a candidate therapeutic agent in laryngeal carcinoma as it could enhance the sensitivity of cancer cells to radiotherapy [112].

**MicroRNA-206**

Downregulation of microRNA-206 has been reported in breast carcinoma, leiomyoma, lung carcinoma, renal cell carcinoma, and rhabdomyosarcomas [113–117]. In laryngeal carcinoma tissues, microRNA-206 expression was significantly reduced in comparison with the normal laryngeal tissues [118, 119]. Zhang et al. demonstrated that the expression levels of miR-206 are associated with the T grade, nodal metastasis and clinical stage of patients with laryngeal carcinoma [118]. MicroRNA-206 controls the expression of vascular endothelial growth factor (VEGF) in laryngeal cancer cells. VEGF is a prognostic indicator of laryngeal carcinoma and is correlated with tumor size and lymph node metastasis [118, 120]. Liu et al. show that miR-206 controls cell migration and invasion through modulating the actin cytoskeleton [121]. MicroRNA-206 could induce apoptosis and inhibit the antiapoptotic pathways such as notch3 [113]. Taken together, miR-206 is suggested to be a candidate tumor-suppressing microRNA in human cancers.

#### 2.5.2. Candidate Oncogenic MicroRNA in Laryngeal Carcinoma

In laryngeal carcinoma, studies on tumor-suppressing microRNA are comparably more than the oncogenic microRNA. One representative example of the oncogenic microRNA identified in laryngeal carcinoma is microRNA-21. MicroRNA-21 upregulation was first reported in human glioblastoma in 2005 [122]. Overexpression of microRNA-21 has been reported in numerous human malignancies suggesting its importance in the genesis of cancer. MicroRNA-21 overexpression is associated with cancer at advanced stages and might have both diagnostic and prognostic values [123–125]. Suppressing microRNA-21 expression in laryngeal carcinoma cell could induce apoptosis and prevent cancer cell invasion by inducing cell cycle arrest [126].

#### 2.5.3. Tumor-Suppressing MicroRNA in Laryngeal Carcinoma—Loss through DNA Methylation

MicroRNA is a kind of epigenetic regulator. MicroRNA itself however is also controled by epigenetic mechanisms including DNA methylation.

**Methylated MicroRNA-9**

According to microRNA registry miRBase (http://www.mirbase.org/), microRNA 9 has 3 isoforms. They are hsa-mir-9-1 (MI0000466), hsa-mir-9-2 (MI0000467), and hsa-mir-9-3 (MI0000468). In oral and oral pharyngeal carcinoma, microRNA-9 is a candidate tumor suppressor and could control cell proliferation through regulating the PTEN pathway. MicroRNA-9 methylation has recently been reported in oral pharyngeal carcinoma including laryngeal carcinoma [127]. Methylated microRNA-9 could differentiate laryngeal cancer from adjacent histologically normal tissues with high specificity (ranging from 97 to 100%).

**Methylated MicroRNA-137**

Downregulation of microRNA-137 has been reported in head and neck malignancies [128]. The association between microRNA-137 downregulation and microRNA-137 methylation has been reported in a number of solid cancers including head and neck squamous cell carcinoma [129]. Head and neck squamous cell carcinoma patients with methylated microRNA-137 have poor survival rate [129]. In glioblastoma multiforme cells, expression of microRNA-137 could inhibit cancer cell proliferation [130]. Downregulation of microRNA-137 by DNA methylation is an early event in colorectal cancer [131]. Balaguer suggested that methylated microRNA-137 might have a therapeutic use as reverse expression of microRNA-137 in colorectal cancer cell could inhibit proliferation of colorectal cancer cells [131]. In laryngeal carcinoma, however, the functional roles of microRNA-137 remained to be elucidated.

#### 2.5.4. Midsize Noncoding RNA Associated with Laryngeal Carcinoma

Small nucleolar RNA is involved in the modification and stability of ribosomal RNA [132]. Although its function remains to be clarified, differential expression of small nucleolar RNA is observed in non-small-cell lung cancer and could be employed in cancer screening. In addition, the highly expressed small nucleolar RNA could be detected in the peripheral blood of cancer patients suggesting that it may be useful to be used as a kind of molecular marker for cancer patients [133]. Recently, Mirisola et al. performed a genome-wide gene expression profiling of 20 laryngeal carcinoma and showed that 2 small noncoding RNAs, SNORA16A and SNORD14C, were associated with the risk of laryngeal carcinoma development [134].

## 3. Conclusion

Increasing evidence suggests that epigenetic alteration is playing a critical part in the development of head and neck carcinoma. In comparison with squamous cell carcinoma of other head and neck regions, studies on the epigenetic dysregulation on laryngeal carcinoma are relatively fewer and most studies focused on a few of the epigenetic changes in their sample group. With the recent advance in technology such as next-generation sequencing and microarray (e.g., methylation array and microRNA array), we could foresee that epigenetic studies on laryngeal carcinoma will change from candidate gene approach to global high-throughput profiling. This provides an opportunity to explore and dissect the disease from different perspectives. More importantly, suitable epigenetic markers could be identified through the gaining understanding of the disease.
